# ATP-dependent thermoring basis for the heat unfolding of the first nucleotide-binding domain isolated from human CFTR

**DOI:** 10.21203/rs.3.rs-5479740/v1

**Published:** 2024-11-21

**Authors:** Guangyu Wang

**Affiliations:** University of California School of Medicine, Davis

**Keywords:** cooperative misfolding, grid thermodynamic signature, noncovalent structure, least-stable interaction, partial unfolding, melting threshold

## Abstract

Traditionally, the thermostability of a protein is defined by a melting temperature, at which half of the protein is unfolded. However, this definition cannot indicate the structural origin of a heat-induced unfolding pathway. Here, the thermoring structures were studied on the ATP-dependent heat-induced unfolding of the first nucleotide-binding domain from the human cystic fibrosis transmembrane conductance regulator. The results showed that initial theoretical and experimental melting thresholds aligned well after three structural perturbations including the F508del mutation, the most common cause of cystic fibrosis. This alignment further demonstrated that the heat-induced unfolding process began with the disruption of the least-stable noncovalent interaction within the biggest thermoring along the single peptide chain. The C-terminal region, which was related to the least-stable noncovalent interaction and the ATP-dependent dimerization of two nucleotide-binding domains, emerged as a crucial determinant of the thermal stability of the isolated protein and a potential interfacial drug target to alleviate the thermal defect caused by the F508del mutation. This groundbreaking discovery significantly advances our understanding of protein activity, thermal stability, and molecular pathology.

## INTRODUCTION

Proteins generally unfold above the basal temperature of the organism in which they evolved. In some missense mutations, unfolding transitions induce protein denaturation in tertiary or secondary structure and/or toxic aggregation, leading to extensive, serious and prevalent human diseases (1–15). However, the origin of the heat unfolding pathway is still elusive because different biophysical measurements arrive at distinct conclusions. A good example is the most common cystic fibrosis-causing mutation F508del in the first nucleotide binding domain (hNBD1) from the human cystic fibrosis transmembrane conductance regulator (hCFTR) at the apical cell surface of epithelia (16).

CFTR is a multi-domain membrane protein, consisting of transmembrane domain 1 (TMD1) and 2 (TMD2), nucleotide binding domains 1 (NBD1) and 2 (NBD2) and a unique regulatory (R) domain between NBD1 and TMD2. Despite belonging to the family of ATP-binding cassette (ABC) transporters, it functions as an ATP-gated anion channel. Upon phosphorylation of the R domain, the ATP-dependent NBD dimerization allows a conformational wave to extend to TMD1 and TMD2 through swapping interactions between NBD1 or NBD2 and intracellular loop 4 (ICL4) or 1 (ICL1), respectively, for channel opening (17–18).

When the regulatory insert (RI) (405–436) and extension (RE) (647–678) are deleted to promote the formation of a head-to-tail homodimer and the biogenesis of F508del-CFTR in tissue culture cells (19–21), hNBD1-Δ(RI,RE) (387–646(Δ405–436)) exhibits various melting thresholds in the presence of 0.125 mM ATP and 10% glycerol (a molecular chaperone) to increase stability (22). Although circular dichroism (CD) at 230 nm (far-UV wavelength region) indicates a significant melting temperature threshold (${\text{T}}_{\text{m}}$) of 51°C, CD at 297 nm (near-UV wavelength region) and the Trp fluorescence signal (excitation wavelength at 290 nm and emission wavelength at 340 nm) reveal it as 45°C, which is consistent with the differential scanning calorimetry (DSC) unfolding curve (22).

Similarly, when F508 is also deleted, (F508del)hNBD1-Δ(RI,RE) has a T_m_ of 46°C for far-UV CD but a ${\text{T}}_{\text{m}}$ of 40°C for near-UV CD, DSC and intrinsic Trp fluorescence assays. These results demonstrate that heat unfolding of hNBD1-Δ(RI,RE) with or without F508 actually starts with a partial change in tertiary structure, leading to an ATP-free aggregation-prone “molten globule” intermediate in the presence of physiological concentrations of ATP (22).

These observations are reminiscent of partial heat-induced unfolding in the tertiary structure, which leads to irreversible inactivation from a pre-open closed state and subsequent aggregation in rat transient receptor potential vanilloid-1 (rTRPV1) upon the release of phosphatidylinositol (PI) from the active vanilloid site (23–24). Although the 3D crystal structures of hNBD1-Δ(RI,RE) with or without F508 at 8°C have been available, and stability prediction for mutations in hNBD1 has shown some computational success (25), the precise structural motifs or thermodynamic signatures for the initial heat unfolding are still unknown,

Recently, a graph theory approach has been developed to introduce a novel concept, model, direction, and feasibility for understanding the temperature sensitivity of biomacromolecules. By mapping networks of temperature-dependent noncovalent interactions such as H-bonds, salt bridges and π interactions from high-resolution 3D structures, well-organized fluidic-like grids of various sizes can be comprehensively constrained and defined as thermo-sensitive rings or thermorings (23, 26–31). Since each thermoring contributes to the distinct structural or functional trait of the given proteins in response to various temperatures, the specifc localized grid-based thermorings can be identified as structural motifs or thermodynamic signatures for initial heat unfolding.

In this study, the thermoring structures of hNBD1 with Mg/ATP bound were analyzed in response to various site-mutation perturbations. Although the deletion of F508 decreased the calculated melting temperature threshold (${\text{T}}_{\text{m}}$) of hNBD1, the subsequent deletion of (RE, RI) or three second-site suppressor mutations (3S, F429S/F494N/Q637R) increased it. Since the calculated ${\text{T}}_{\text{m}}$ values of the biggest thermorings to disrupt the least-stable noncovalent interactions in all the hNBD1 constructs matched the initial experimental ones, heat unfolding of hNBD1 may start with the melting of the least-stable noncovalent interaction in the biggest thermoring along the single peptide chain. Notably, both the least-stable noncovalent interactions and the head-to-tail dimerization of NBD1-NBD1 were closely related to the C-terminal region of hNBD1 before the 3S mutations, rendering that region a crucial determinant of the thermal stability of hNBD1 and a potential drug target to resuce the interfacial thermal defect of the F508del mutant.

## Results

### Identification of the biggest thermoring with a corresponding melting threshold in hNBD1

**Figure 1:**
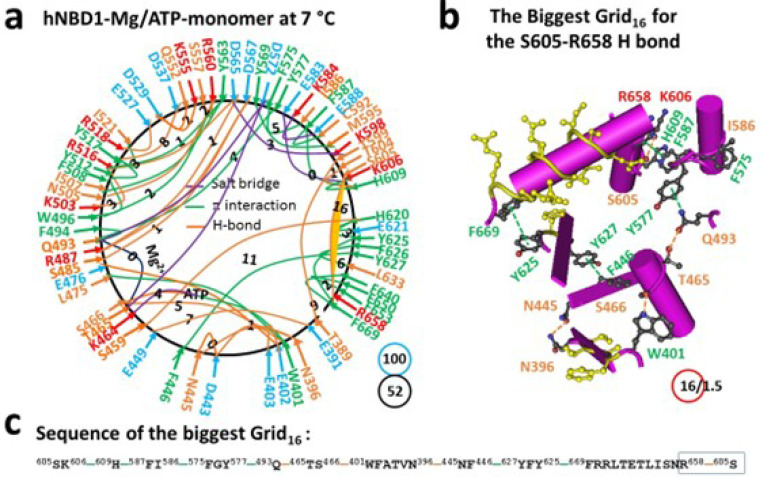
The thermoring structures of hNBD1 with F508 in the Mg/ATP bound state at 7 °C. (**a**) The grid-like noncovalently interacting mesh network based on the X-ray structure of an isolated hNBD1 with F508 in a Mg/ATP bound state at 7 °C (PDB ID, 2BBO, 2.55 Å). Salt bridges, H-bonds and p interactions are colored purple, orange, and green, respectively. The constrained grid sizes required to control the least-stable noncovalent interactions in the grids are labeled with black numbers. The least-stable S605-R658 H-bond in the biggest Grid_16_ is highlighted. The total grid sizes and the total grid size-controlled noncovalent interactions along the single peptide chain from T389 to P676 are shown in cyan and black circles, respectively. (**b**) The structure of the biggest Grid_16_ with a 16-residue size at the RE/NBD1 interface to control the least-stable S605-R658 H-bond. The grid size and the equivalent basic H-bonds for the least-stable noncovalent interaction are shown in and near a red circle. (**c**) The sequence of the biggest Grid_16_ to control the least-stable S605-R658 H-bond in the blue box.

The isolated hNBD1 is a monomer in the presence of 2–5 mM Mg/ATP and 5% glycerol, which prevents hNBD1 aggregation. This single peptide spans from S388 to S678. The X-ray structure (PDB, 2BBO) reveals that the regulatory insertion (RI) (F405-L435) is disordered (32). With Mg/ATP bound to hNBD1, Mg^2+^ connected T465, Q493 and D572 together, creating the smallest thermoring with a zero-residue size. Meanwhile, ATP linked W401, K464, S466 and L475 together (Figs. 1a, S1). Even without other hCFTR domains, F508 still formed a strong core grid mesh network through W496-F508, Y507-Y563-R516 and Y517-Y512 π bridges, as well as the R516-N505 and Y512-K503 H-bonds. With a total of 52 noncovalent interactions and 100 grid sizes, the calculated systematic thermal instability (${\text{T}}_{\text{i}}$) was 1.92 (Table 1).

Notably, in the presence of the regulatory extension (RE, C647 to S678), the biggest Grid_16_ appeared to control the S605-R658 H-bond through the thermo-sensitive ring from S605 to K606, H609, F587, I586, F575, Y577, Q493, T465, S466, W401, N396, N445, F446, Y627, Y625, F669, R658 and back to S605 (Fig. 1b-c). When sealed with 1.5 equivalent basic H-bonds, the calculated T_m_ was approximately 37°C, matching the melting threshold measured by intrinsic tryptophan fluorescence or differential scanning fluorimetry (DSF) (Table 1) (33–36).

### Removal of F508 from hNBD1 regulates the biggest thermoring with a decreased melting threshold

**Figure 2:**
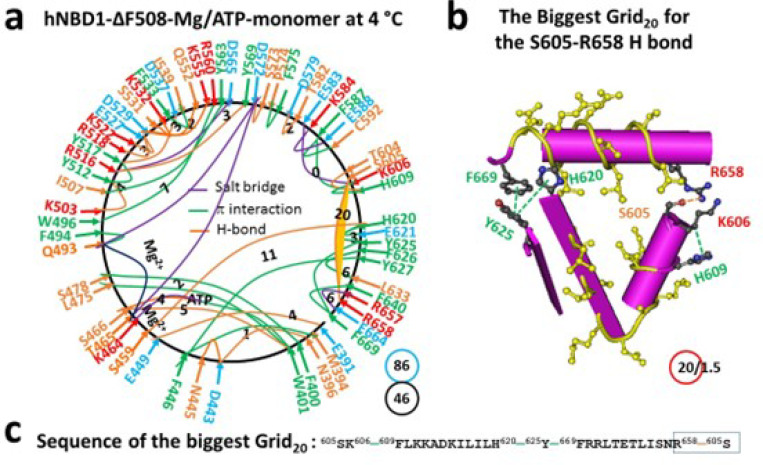
The thermoring structures of hNBD1 without F508 in the Mg/ATP bound state at 4 °C. (**a**) The grid-like noncovalently interacting mesh network based on the X-ray structure of an isolated NBD1 without F508 in a Mg/ATP bound state at 4 °C (PDB ID, 1XMJ, 2.3 Å). Salt bridges, H-bonds and p interactions are colored purple, orange, and green, respectively. The constrained grid sizes required to control the least-stable noncovalent interactions in the grids are labeled with black numbers. The least-stable S605-R658 H-bond in the biggest Grid_20_ is highlighted. The total grid sizes and the total grid size-controlled noncovalent interactions along the single peptide chain from E391 to A675 are shown in cyan and black circles, respectively. (**b**) The structure of the biggest Grid_20_ with a 20-residue size at the RE/NBD1 interface to control the least-stable S605-R658 H-bond. The grid size and the equivalent basic H-bonds for the least-stable noncovalent interaction are shown in and near a red circle. (**c**) The sequence of the biggest Grid_20_ to control the least-stable S605-R658 H-bond in the blue box.

F508 is positioned in the α-helical (ABCα) subdomain of hNBD1 (19, 37). When F508 was deleted to disconnect the W496-F508 π interaction, although the protein was still a monomer and had the same Mg/ATP binding site and similar unstructured RI (E403-G437), a conformational change expanded from N505 to both T389 and E664 through networks of side-chain interactions (Figs. 2a, S2). For example, when the N505-R560 H-bond was broken, the D565-K598 salt bridge, the Q493-F577, Y569-M595 and F575-I586 π interactions, and the D565-R487-D567-S485 and T389-T599 H-bonds were also disconnected. Meanwhile, the R657-E664 salt bridge and the M394-F446 and F400-S478 π interactions were formed (Figures S1, S2). As a result, cooperative misfolding decreased the total noncovalent interactions and the total grid sizes from 52 and 100 to 46 and 86, respectively, leading to a slight decrease in systematic thermal instability (${\text{T}}_{\text{i}}$) from 1.92 to 1.87 (Table 1).

On the other hand, while the S605-R658 H-bond remained the least-stable noncovalent interaction along the single peptide chain, the biggest thermoring shifted from Grid_16_ to Grid_20_ (Fig. 2a). It had a 20-residue size via the thermoring from S605 to K606, F609, H620, Y625, F669, R658 and back to S605 (Fig. 2b-c). For 1.5 equivalent basic H-bonds to secure it, the calculated ${\text{T}}_{\text{m}}$ was about 29°C, which closely matched the melting threshold of 30°C measured by intrinsic tryptophan fluorescence or DSF (Table 1) (33–36). In this regard, a decrease in the ${\text{T}}_{\text{m}}$ rendered the (F508del)hNBD1 unstable, reminiscent of the destabilized A149P, the most prevalent mutant aldolase B associated with hereditary fructose intolerance (2).

### Deletion of RE from hNBD1 alters the biggest thermoring with an increased melting threshold

If the biggest Grid_16_ determines the melting threshold of hNBD1 via the least-stable S605-R658 H-bond, deleting RE should disrupt this H-bond in the biggest thermoring and thus increase the melting threshold. To test this hypothesis, the thermoring structure of hNBD1 without RI and RE was examined. Since RI is typically unstructured in hNBD1, removing RE is expected to directly impact the biggest thermoring for an increased melting threshold.

**Figure 3:**
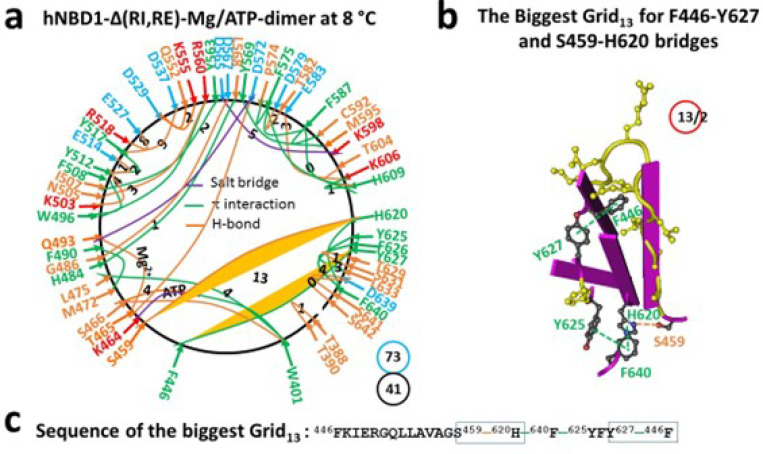
The thermoring structures of hNBD1-D(RE,RI) with F508 in the Mg/ATP bound state at 8 °C. (**a**) The grid-like noncovalently interacting mesh network based on the X-ray structure of an isolated NBD1-D(RE,RI) with F508 in a Mg/ATP bound state at 8 °C (PDB ID, 2PZE, 1.7 Å). Salt bridges, H-bonds and p interactions are colored purple, orange, and green, respectively. The constrained grid sizes required to control the least-stable noncovalent interactions in the grids are labeled with black numbers. The least-stable S459-H620 H-bond and Y627-F446 p interaction in the biggest Grid_13_ are highlighted. The total grid sizes and the total grid size-controlled noncovalent interactions along the single peptide chain from S386 to M645 are shown in cyan and black circles, respectively. (**b**) The structure of the biggest Grid_13_ with a 13-residue size at the interface between N- and C- termini to control the least-stable S459-H620 H-bond and Y627-F446 p interaction. The grid size and the equivalent basic H-bonds for the least-stable noncovalent interactions are shown in and near a red circle. (c) The sequence of the biggest Grid_13_ to control the least-stable S459-H620 H-bond and Y627-F446 p interaction in the blue box.

When RE was deleted from the hNBD1 dimer, the S605-R658 H-bond and the biggest Grid_16_ were both dissociated, along with the disappearance of the nearby F650-F653, F669-F640/Y625 and H620-E621 π interactions, and the appearance of the nearby F640-Y625/H620 π interactions and the T629-S631 and D639-S641/S642 H-bonds (Figs. 3a, S3). As this conformational change transmitted, some noncovalent interactions were no longer present. For example. the E588-K584 salt bridge, the S485-D567-R487-D565-R516, E476-E402, E449-E391, and N445/D443-N396 H-bonds, and the F575-I586, Y577-Q493, and Y563-R516 π interactions were absent. Meanwhile, other noncovalent interactions such as the L568-H484 and F490-M472 π interactions were present. Notably, the side chain of D567 formed an H-bond with the backbone NH at G486 neat the H484-L568 π interaction (Figures S1 and S3).

Taken together, although the Mg/ATP binding site was still maintained in favor of the formation of the dimer, both the total noncovalent interactions and the total grid sizes decreased from 52 and 100 to 41 and 73, respectively. Thus, the systematic thermal instability $\left({\text{T}}_{\text{i}}\right)$ significantly decreased from 1.92 to 1.78 (Table 1). When the biggest Grid_16_ disappeared upon the RE removal, the new biggest Grid_13_ appeared, linking the C and N termini together (Fig. 3a). It had a 13-residue size to control the F446-Y627 π-π interaction and the S459-H620 H-bond via the thermoring from F446 to S459, H620, F640, Y625, Y627 and back to F446 (Fig. 3b-c). With 2 equivalent basic H-bonds to seal it, the calculated ${\text{T}}_{\text{m}}$ was about 48°C, matching the melting threshold of 48°C measured by DSC (Table 1) (20, 22). Thus, the RE deletion did increase the ${\text{T}}_{\text{m}}$ but decrease the ${\text{T}}_{\text{i}}$.

### Deletion of F508 from hNBD1-Δ(RI-RE) further adjusts the biggest thermoring with a decreased melting threshold

**Figure 4:**
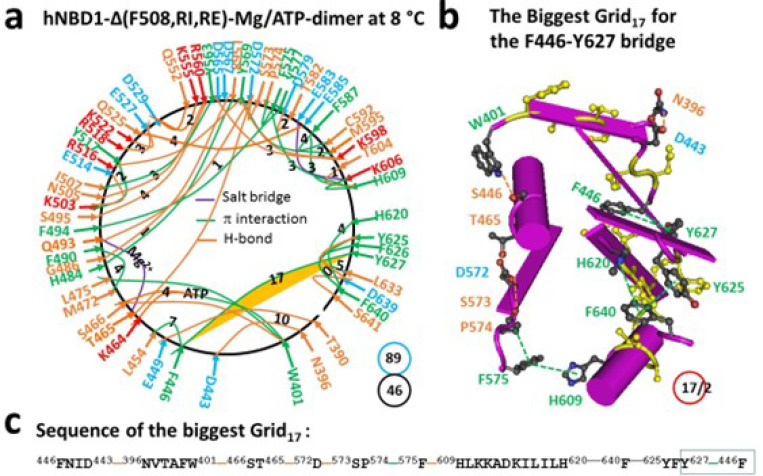
The thermoring structures of hNBD1-D(RE,RI) without F508 in the Mg/ATP bound state at 8 °C. (**a**) The grid-like noncovalently interacting mesh network based on the X-ray structure of an isolated NBD1-D(RE,RI) without F508 in a Mg/ATP bound state at 8 °C (PDB ID, 2PZF, 2.0 Å). Salt bridges, H-bonds and p interactions are colored purple, orange, and green, respectively. The constrained grid sizes required to control the least-stable noncovalent interactions in the grids are labeled with black numbers. The least-stable Y627-F446 p interaction in the biggest Grid_17_ is highlighted. The total grid sizes and the total grid size-controlled noncovalent interactions along the single peptide chain from S386 to G646 are shown in cyan and black circles, respectively. (**b**) The structure of the biggest Grid_17_ with a 17-residue size at the interface between N and C termini to control the least-stable Y627-F446 p interaction. The grid size and the equivalent basic H-bonds for the least-stable noncovalent interaction are shown in and near a red circle. (**c**) The sequence of the biggest Grid_17_ to control the least-stable Y627-F446 p interaction in the blue box.

When F508 was further removed from hNBD1-**Δ**(RE, RI), the Mg/ATP binding site remained intact for the formation of the dimer. However, there was a global conformational change (Fig. 4a). For example, the S459-H620, Y512-E514, Y517-D537, R629-S631 and D639-S642 H-bonds, as well as the F508-W496-R560, K503-Y512-Y517 and F575-F587 π interactions were removed. On the other hand, the T390-E449, N396-D443, S495-R560, K503-E514, Q525-E585, Y569-K598 and Y625-D639 H-bonds were added (Figures S3, S4). As a result, the biggest Grid_13_ turned to Grid_17_ to maintain the same F446-Y627 π−π interaction. It then had a 17-residue size via the thermoring from F446 to D443, N396, W401, S466, T465, D572, S573, P574, F575, H609, H620, F640, Y625, Y627, and back to F446 (Fig. 4b-c). Thus, a single amino acid deletion disrupted functional networks throughout the protein, leading to protein misfolding. This is reminiscent of phenylalanine hydroxylase (PAH) missense mutations, which induce conformational protein destabilization and loss of PAH function (4).

Due to the additional CH-π interaction between F446 and Y627, this biggest thermoring was actually sealed by 2.0 equivalent basic H-bonds. In this case, the calculated ${\text{T}}_{\text{m}}$ was about 40°C, matching the initial melting threshold measured by DSC (Table 1) (22). Additionally, both the total noncovalent interactions and the total grid sizes significantly increased from 41 and 73 to 46 and 89, respectively. Therefore, the systematic thermal instability $\left({\text{T}}_{\text{i}}\right)$ also increased from 1.78 to 1.93, along with the decrease in the ${\text{T}}_{\text{m}}$ from 48°C to 40°C (Table 1).

### Three second-site suppressor mutations (3S) also increase the melting threshold of hNBD1-F508del via an alternative biggest thermoring

**Figure 5:**
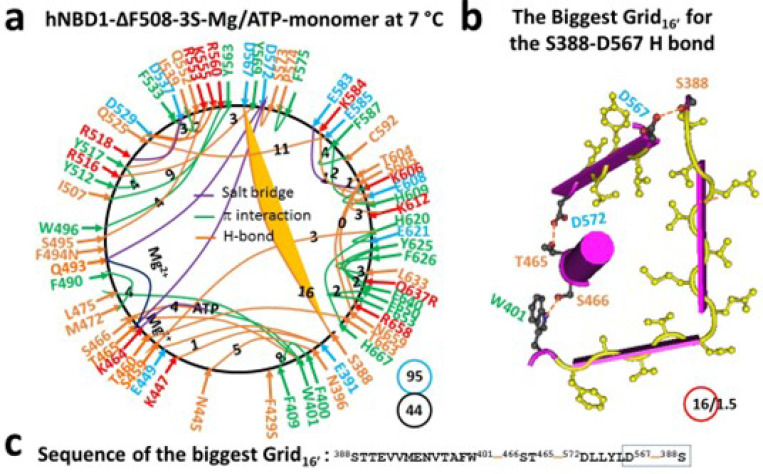
The thermoring structures of hNBD1–3S without F508 in the Mg/ATP bound state at 7 °C. (**a**) The grid-like noncovalently interacting mesh network based on the X-ray structure of an isolated hNBD1-F429S/F494N/Q637R without F508 in a Mg/ATP bound state at 7 °C (PDB ID, 2BBS, 2.05 Å). Salt bridges, H-bonds and p interactions are colored in purple, orange, and green, respectively. The constrained grid sizes required to control the least-stable noncovalent interactions in the grids are labeled with black numbers. The least-stable S388-D567 H-bind in the biggest Grid_16_, is highlighted. The total grid sizes and the total grid size-controlled noncovalent interactions along the single peptide chain from S388 to L671 are shown in cyan and black circles, respectively. (**b**) The structure of the biggest Grid_16_, with a 16-residue size to control the least-stable S388-D567 H-bind. The grid size and the equivalent basic H-bonds for the least-stable noncovalent interaction are shown in and near a red circle. (**c**) The sequence of the biggest Grid_16_, to control the the least-stable S388-D567 H-bind in the blue box.

In addition to the deletion of RE and RI, three second-site suppressor mutations such as F429S/F494N/Q637R (3S) are also used to counteract the destabilizing effect of the F508del mutation by increasing the thermo-stability of (F508del)hNBD1 (38). F429S, F494N and Q637R are located in the RI, near the Mg^2+^ site and the RE, respectively (Fig. 5a) (32). Among them, F494 formed a π interaction with Q493 while F429 and Q637 were silent in (F508del)hNBD1 (Fig. 2a). However, their mutations significantly rearranged the entire conformation in a peptide range from S388 to H667 (Figs. 5a, S5). First, near the F429S mutation site, the M394-F446-Y627 and F400-S478 π interactions and the N396-D443-N445 H-bonds were disrupted but the E391-K447 and N396-N445 H-bonds were present; Second, near the F484N mutation site, the K503-Y512 and R518/K522-E527 H-bonds and the R516-D565 salt bridge were replaced with the S495-R553 and Q525-E585 H-bonds and the R518-E537 salt bridge; Third, near the Q637R mutation site, the H620-Y625-F669-F640 π interactions were substituted by the H620-H667 and Y625-R637/F640 π interactions (Figures S2, S5).

Of special note, S605 H-bonded with not only R658 but also N659, eliminating the previous biggest Grid_20_. Meanwhile, the K584-E588 salt bridge moved to the K584-E608 H-bond (Figs. 2a and 5a). Taken as a whole, a new biggest thermoring was identified as Grid_16_, (Fig. 5b-c). It had a 16-residue size to control the least-stable S388-D567 H-bond via the thermoring from S388 to W401, S466, T465, D572, D567, and back to S388. Once 1.5 equivalent basic H-bonds sealed it, the calculated melting threshold (${\text{T}}_{\text{m}}$) was about 37°C, which was the same as the melting threshold measured by DSC in the presence of 10% glycerol and 5 mM ATP (Table 1) (20). Thus, the 3S mutations alternatively increased the melting threshold from 29°C to 37°C. However, the total noncovalent interactions and total grid sizes changed from 46 and 86 to 44 and 95, respectively (Figs. 2a, 5a). Thus, the systematic thermal instability (${\text{T}}_{\text{i}}$) did not lower but raised from 1.87 to 2.16 (Table 1).

## Discussion

The folding pathway of hNBD1 is critical for understanding the molecular pathology of the most common CF-causing F508del mutation in the tertiary noncovalent structure of the full-length CFTR anion channel. In this study, the thermoring structures of the isolated hNBD1 construct were analyzed under various structural perturbations. While three smaller thermorings were highly conserved, possibly to maintain the overall secondary structures, a rearrangement of the thermoring structures was observed along the entire single peptide chain following the deletion of F508, RE, RI or the nearby 3S mutations. These manipulations resulted in different biggest thermorings with matched melting thresholds, necessary for initiating the heat unfolding of various hNBD1 constructs. No matter whether F508 was deleted from hNBD1 or hNBD1-Δ(RE, RI), along with the decrease in the melting threshold, the systematic thermal instability (${\text{T}}_{\text{i}}$) was also increased. Furthermore, the C-terminal region, closely associated with the least-stable noncovalent interactions and the head-to-tail dimerization of NBD1-NBD1 prior to the 3S mutations, may play a critical role in stabilizing the hNBD1 and the drug development.

### Melting of the biggest thermorings are required for the initial heat unfolding of hNBD1

Previous studies have shown that the melting threshold of a given protein is influenced by the size of the biggest thermoring and the strength of the least-stable noncovalent interaction within it (26–30). In this study, the matched melting thresholds further demonstrated that the melting of the biggest thermorings was necessary for the initial heat unfolding of hNBD1. In the presence of 2–5 mM ATP and 5% glycerol, the hNBD1 construct with RE and RI displayed the biggest Grid_16_, which governed the least-stable S605-R658 H-bond, resulting in a matched melting threshold of 37°C (Fig. 1, Table 1). Although the deletion of F508 did not affect the least-stable S605-R658 H-bond along the single peptide from T389 to F669, the biggest thermoring changed from Grid_16_ to Grid_20_ following global cooperative misfolding (Fig. 2), leading to an increase in size from 16 to 20 and a decrease in the melting threshold from 37°C to 29°C (Table 1).

In agreement with this proposal, the disruption of the least-stable S605-R658 H-bond upon the deletion of RE also altered the biggest thermoring, leading to an increase in the melting threshold (Figs. 1–4). For the full-length NBD1, in the presence of 2–5 mMATP and 5% glycerol, the biggest Grid_13_ controlled the least-stable F446-Y627 and S459-H620 bridges, resulting in a matched melting temperature of 48°C (Fig. 3, Table 1). Similarly, with the RE removal disrupting the S605-R658 H-bond, the hNBD1-F508del also changed the biggest thermo-ring from Grid_20_ to Grid_17_ to control the F446-Y627 π interactions, causing an increase in the melting temperature from 29°C to 40°C (Fig. 4, Table 1). In any way, the deletion of F508 lowered the melting temperature threshold by 8°C, which aligned with the calculated difference of 8°C (Table 1) (33–36).

**Figure 6:**
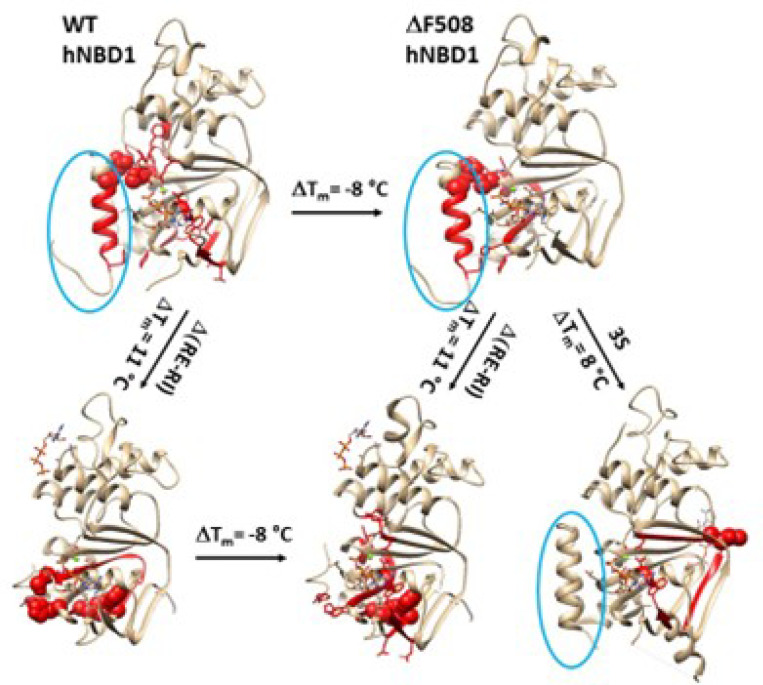
Effects of DRE and 3S mutations on the biggest thermorings in hNBD1 with or without F508. The x-ray structures of hNBD1 with (2BBO) and without F508 (1XMJ), hNBD1- D(RE, RI) with (2ZPE) and without F508 (2PZF), and hNBD1–3S without F508 (2BBS) are used for the models. RE is circled in blue and the biggest thermorings are shown in red. The residues responsible for the least-stable noncovalent interactions in the biggest thermorings are shown in space fills.

Similarly, the 3S mutations in or near the RI and RE and the Mg^2+^ site also affected the nearby conformation, leading to a change in the biggest thermoring from Grid_20_ to Grid_16_,for the increased melting threshold of 37°C (Fig. 5, Table 1) (20). Overall, all these matched melting thresholds were measured by DSF or DSC, in agreement with intrinsic Trp fluorescence or the CD at near-UV rather than far-UV (Table 1) (20, 22, 36). Thus, the global cooperative unfolding and refolding of noncovalent structures in hNBD1 under various structural perturbations factually dictates the biggest thermorings for matched melting thresholds (Fig. 6). In fact, when disease-causing missense mutations in actin binding domain 1 of dystrophin exhibited a non-cooperative heat unfolding transition, only the melting thresholds are available to evaluate the thermostability of the missense mutants (6). Accordingly, the melting temperature threshold (${\text{T}}_{\text{m}}$) could better characterize the thermal stability of a protein.

### Overall secondary structures are conserved during the initial melting of the biggest thermorings

**Figure 7:**
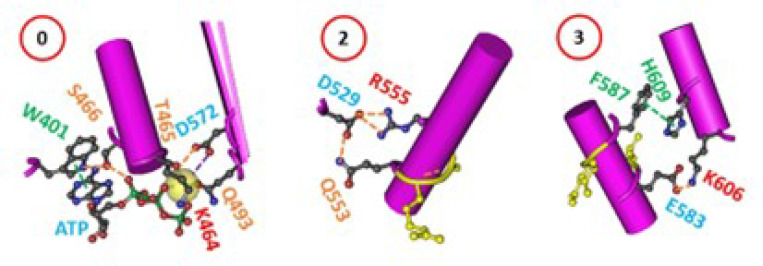
Smaller highly conserved thermorings in hNBD1 upon structural perturbations. The X-ray structure of hNBD1-F508del with the 3S mutations (2BBS) was chosen for the models. The thermoring sizes are circled in red.

In addition to the biggest thermorings for the matched melting thresholds of hNBD1, three smaller thermorings were found to be highly conserved in hNBD1 upon the removal of F508, (RE, RI) or the 3S mutations (Fig. 7). The first was the Mg/ATP binding site, consisting of D572, T465, Q-loop Q493 and the phosphate group of ATP, forming the smallest thermoring with a zero-residue size for the Mg^2+^ site. The second, related to the signature LSGGQ half-site in hNBD1 (20), was the smaller Grid_2_ formed by the thermoring from D529 to Q552, R555, and back to D529. The third was the smaller Grid_3_ lined by the thermoring from E583 to Y587, H609, K606, and back to E583. These smaller thermorings may act as stable anchors for the overall stability and integrity of the secondary structure and ATP binding at the Walker and signature site (39). A similar scenario has been observed for disease-causing actin-binding domain 1 (ABD1) mutants of dystrophin, which retained near WT affinity for actin filaments (6). Thus, it is reasonable that these residues are closely linked to cystic fibrosis with mutations such as D529H/G, Q552K/X, R555G, F587I, H609L/R, D572N, and Q493X/P/R (http://www.genet.sickkids.on.ca/cftr).

Traditionally, heat-induced unfolding of a ligand-free protein follows a two-state model that involves changes in both tertiary and secondary structures (40–41). However, this is not always the case when Mg/ATP is bound to hNBD1 at physiological concentrations. In this study, the heat-induced melting of the biggest thermoring in hNBD1 with or without F508 disrupted the least-stable S605-R658 H-bond (Figs. 1–2). When the (RE, RI) deletion also disrupted this H-bond (Figs. 3–4), the overall secondary structures were conserved (Fig. 6). Therefore, the heat-induced disruption of the same least-stable noncovalent interaction in the biggest thermoring Grid_16_ or Grid_20_ may only induce a conformational rearrangement in tertiary structure rather than secondary structure. Small perturbations in the far-UV CD curves of hNBD1-Δ(RI,RE) and (F508del)hNBD1-Δ(RI,RE) at temperature lower than 51°C and 46°C, respectively, may be due to the disruption of the π interaction between juxtaposed aromatic residues F446 and Y627 (2, 22, 26, 42). In addition, when the least-stable S605-R658 H-bond was disrupted by the removal of RE, the Q493-Y577 and Q493-F494 π interactions near the Mg/ATP site of hNBD1 and (F508del)hNBD1 were also broken (Figs. 1–4). This may account for the weakened Mg/ATP affinity upon the heat-induced melting of the least-stable S605-R658 H-bond in the biggest thermorings Grid_16_ and Grid_20_ (22).

Given that the F508del mutation promotes molten globule formation of hNBD1-Δ(RE, RI) but ATP and the 3S mutations prohibit it at human body temperature of 37°C even in the presence of physiological concentrations of ATP (22, 43), the S459-H620 H-bond, preserved in hNBD1-Δ(RE, RI) and the 3S mutations but absent in (F508del)hNBD1 (Figs. 3–5), may take a pivotal role in stabilizing the Mg/ATP binding and the native conformation of hNBD1.

### Removal of F508 increases the systematic thermal instability of hNBD1-Δ(RE, RI)

For the full-length hCFTR anion channel, the RE and RI regions are disordered (44–45). Therefore, it is interesting to evaluate the effects of the F508 deletion on the systematic thermal instability (${\text{T}}_{\text{i}}$) of the isolated hNBD1-Δ(RE, RI). For the wild-type (WT) hNBD1, the (RE, RI) deletion significantly decreased the ${\text{T}}_{\text{i}}$ from 1.92 to 1.78. However, the F508 deletion dramatically raised the ${\text{T}}_{\text{i}}$ of hNBD1-Δ(RE, RI) from 1.78 to 1.93 (Table 1). These results were consistent with the increased conformational flexibility of (F508del)hNBD1 (34), which favors the formation of the molten globule intermediate and thus renders the mutant susceptible to misfolding in response to physical or chemical stress (22;43).

### C-terminal region is the determinant of thermo-stability of hNBD1

The missense mutation in some human diseases can destabilize the native conformation of protein globally, resulting in a partial unfolding and aggregation in response to a thermal stress. For example, a single F9S mutation in the N-terminal domain of mouse γS-crystallin brings about the severe *Opj* cataract, along with disruption of cellular organization and appearance of fibrillar structures in the lens (5). A similar case was also reported regarding medium-chain acyl-CoA dehydrogenase deficiency (MCADD) caused by mutations in the ACADM gene (8). Therefore, it is necessary to identify the least-stable link for developing a strategy to rescue the thermal defect. In the case of hNBD1, the least-stable noncovalent interaction was the R548-S605 H-bond (Fig. 1). The deletion of F508 did not affect this H-bond (Fig. 2). When RE and RI were deleted, hNBD1-Δ(RI,RE) had the least-stable H-bonding pairs H620-S459 and Y627-F446 (Fig. 3). With F508 deletion, the H620-S459 H-bond disappeared (Fig. 4). Therefore, the C-terminal region may play a critical role in stabilizing hNBD1. Once the 3S mutations introduced a new S605-N659 H-bond to suppress the least-stable S650-R658 H-bond, it is reasonable that the melting threshold increased along with the new least-stable S388-D567 H-bond in the new biggest Grid_16_, (Fig. 5), stabilizing (F508del)hNBD1 (20).

On the other hand, the C-terminal region is located at the NBD1/NBD2 interface and close to the Mg/ATP binding site (Fig. 6). Therefore, any small molecule drug that can enhance the interdomain interface is expected to allosterically stabilize the entire protein and thus rescue the thermal or gating defect of the F508del mutant (34, 46). For example, ATP or dTTP for enhancing the NBD1/NBD2 interface (39, 47), curcumin for gluing the ICL1/ICL4/R interface (48–50), Trikafta (VX770, VX445, and VX661) for strengthening the TMD1/TMD2 interface (44–45). Further mutagenesis is needed to examine the least-stable noncovalent interactions and their effects on hNBD1.

## Conclusions

Many disease-causing missense mutations destabilize the native protein conformation. Therefore, identifying the weakest interaction in these proteins is important for developing a target-selective drug or antibody to enhance thermal stability. Among them, given that the most prevalent cystic fibrosis-causing mutant F508del destabilizes the CFTR channel by affecting the least-stable noncovalent interaction, which was closely related to the C-terminal region near the Mg/ATP site at the interface of NBD1 and NBD2, enhancing the interdomain interface is necessary for rescuing the thermal defect in F508del-hCFTR.

### COMPUTATIONAL METHODS

#### Data mining resources

Five X-ray structures of human NBD1 (hNBD1) with Mg/ATP bound at 4–8 °C were selected for thermoring analysis. They included wild type (WT) hNBD1 (PDB ID, 2BBO, model resolution = 2.55 Å), hNBD1-D(RE,RI) (PDB ID, 2PZE, model resolution = 1.7 Å), (F508del) hNBD1- (PDB ID, 1XMJ, model resolution = 2.3 Å), (F508del)hNBD1-D(RE,RI) (PDB ID, 2PZF, model resolution =2.0 Å), and (F508del)hNBD1–3S (PDB ID, 2BBS, model resolution =2.05 Å) (20, 32, 37).

#### Standard methods for filtering non-covalent interactions

The standard methods for filtering non-covalent interactions, along with precise computation, were the same as previously used, ensuring accurate and repeatable results (24, 26–31). UCSF Chimera was utilized to visualize potential stereo-selective or regio-selective intra-domain lateral noncovalent interactions along the single peptide chain of hNBD1 with or without F508 in the presence or absence of regulatory extension (RE) and insert (RI). These included salt-bridges, H-bonds and lone pair/CH/cation-p interactions between paired amino acid side chains. Detailed cutoff distances and interaction angles were also available in the online Supporting Information (Tables S1, S2, S3, S4, and S5). In this study, approximately at least 40 different noncovalent interactions were identified along the single peptide chain from S386 to G646 or P676 on each protomer.

#### Mapping thermoring structures using the grid thermodynamic model

The established grid thermodynamic model was used to map thermoring structures from the filtered noncovalent interactions (24, 26–31). In brief, along the single peptide chain depicted by the black line from S386 to G646 or P676 of the isolated hNBD1 construct, paired protein residues for a specific noncovalent interaction were represented as nodes and shown as colorful arrows. An adjacency matrix was then created as a systematic fluidic grid-like mesh network with two types of positively curved edges (51). The colorful edge had no free residues between two nodes resulting in a length of zero. When two nodes were connected by a segment of the peptide chain, the length of the black edge was determined by the number of free and silent residues between them. This method allowed each noncovalent interaction to be associated with a subgraph or a constrained topological grid, starting and ending at a specific amino acid residue via the shortest path (geodesic transportation distance) between two connected nodes. A direct path representing the noncovalent interaction had a length of zero. However, the shortest reverse path, unlike the direct path, consisted of the segment of the polypeptide chain and other noncovalent interactions. Based on graph theory and the Floyd-Warshall algorithm (52), the shortest reverse path could be constrained as the minimal total number of free or silent side chains of residues not involved in any noncovalent interactions within a given grid. When the shortest round path length represented the grid size, such a constrained grid could be defined as a thermo-sensitive ring or thermoring denoted as Grid_s_. For example, in the grid-like biochemical reaction mesh network of Figure 1a, the direct path length from N396 to N445 was zero due to an H-bond between them. However, there was another shortest reverse path from N445 to D443 and back to N396 via the N445-D443 and D443-N396 H-bonds. This round path involved no free or silent residues for any non-covalent interactions between side chains, resulting in the creation of thermoring Grid_0_ with a 0-residue size to stabilize the three least-stable H-bonds.

By tracking all thermorings from the biggest grid to the smallest with their respective unshared grid sizes to control thermal unfolding, specific melting temperature thresholds $\left({\text{T}}_{\text{m}}\right)$ could be calculated to identify the least-stable noncovalent interaction or weakest functional link within the given polypeptide chain. Moreover, the total non-covalent interactions and grid sizes along the given polypeptide chain, indicated by black and cyan circles beside the mesh network map, respectively, allowed for the calculation of grid-based systematic thermal instability $\left({\text{T}}_{\text{i}}\right)$.

#### Calculation of the melting temperature threshold $\left({\text{T}}_{\text{m}}\right)$

The same equation used in previous studies on temperature-dependent structures was applied to calculate the melting temperature thresholds $\left({\text{T}}_{\text{m}}\right)$ for thermal unfolding of a specific grid (24, 26–31):

(1) $${\text{T}}_{\text{m}}\left({}^{\circ}\text{C}\right)=34+(\text{n}-2)\times 10+(20-\text{s})\times 2$$

where, n represents the total number of basic H-bonds (each approximately 1 kcal/mol) that are energetically equivalent to the least-stable noncovalent interaction controlled by the given grid; and s is the grid size used to control the least-stable noncovalent interaction in the given grid.

#### Evaluation of the grid-based systemic thermal instability $\left({\text{T}}_{\text{i}}\right)$

The same equation used in previous studies on temperature-dependent structures was utilized to calculate the systematic thermal instability $\left({\text{T}}_{\text{i}}\right)$ along the given polypeptide chain (24, 26–31):

(2) $${\text{T}}_{\text{i}}=\text{S}/\text{N}$$

where, S and N are the total grid sizes and the total non-covalent interactions along a specific polypeptide chain. This calculation allows for evaluation of the protein’s compact conformational entropy.

## Supplementary Material

1

## Figures and Tables

**Table 1:** Grid thermodynamic model-based new parameters induced by heat-unfolding of hNBD1 constructs. The comparative parameters are highlighted in bold.

Construct	hCFTR-NBD1				
PDB ID	2BBO	1XMJ	2PZE	2PZF	2BBS
F508	+	-	+	-	-
3S(F429S/F494N/Q637R)	-	-	-	-	+
(RE, RI)	+	+	-	-	+
Glycerol, %	5	5	10	10	10
ATP, mM	2–5	2–5	5	5	5
Sampling temperature, °C	7	4	8	8	7
Oligmer	monomer	monomer	dimer	dimer	monomer
Name of the biggest grid	Grid_16_	Grid_20_	Grid_13_	Grid_17_	Grid_16_,
grid size (s)	16	20	13	17	16
# of energetically equivalent basic H-bonds (n) controlled by Grid_s_	1.5	1.5	2.0	2.0	1.5
Total non-covalent interactions (N)	52	46	41	46	44
Total grid sizes (S), a.a.	100	86	73	89	95
Systematic thermal instability (${\text{T}}_{\text{i}}$)	1.92	1,87	1.78	1.93	2.16
Calculated ${\text{T}}_{\text{m}}$, °C	**37**	**29**	**48**	**40**	**37**
Measured threshold ${\text{T}}_{\text{m}}$, °C	**37**	**30**	**48**	**40**	**37**
References for measured ${\text{T}}_{\text{m}}$	(33–36)	(33–36)	(20, 22)	(22)	(20)

## Data Availability

All data generated or analyzed during this study are included in this published article and Supporting Information.

## Abbreviations

ABC: ATP-binding cassette
ABD1: actin-binding domain 1
CD: circular dichroism
CFTR: cystic fibrosis transmembrane conductance regulator
DSC: differential scanning calorimetry
DSF: differential scanning fluorimetry
hCFTR: human CFTR
NBD1: the first nucleotide binding domain
hNBD1: human NBD1
ICL1: intracellular loop 1
ICL4: intracellular loop 4
MCADD: medium-chain acyl-CoA dehydrogenase deficiency
PAH: phenylalanine hydroxylase
PI: phosphatidylinositol
R: regulatory
RE: regulatory extension
RI: regulatory insert
${\text{T}}_{\text{i}}$: systematic thermal instability
${\text{T}}_{\text{m}}$: melting temperature threshold
3S: F429S/F494N/Q637R
TMD1: transmembrane domain 1
TMD2: transmembrane domain 2
TRPV1: transient receptor potential vanilloid-1
rTRPV1: rat TRPV1
WT: wild type
