# Supplementary material for: ATP-dependent thermoring basis for the heat unfolding of the first nucleotide-binding domain isolated from human CFTR
Source: Res Sq. 2024 Nov 21:rs.3.rs-5479740. Preprint. [Version 1] doi: 10.21203/rs.3.rs-5479740/v1 (PMC11601864; doi:10.21203/rs.3.rs-5479740/v1)
Supplement: 1 [file NIHPPRS5479740V1-supplement-1.pdf]

Supplementary Files

This is a list of supplementary files associated with this preprint. Click to download.

- SupportingInformationCFTRF508delheatunfolding.pdf
